# Comparing the taxonomic and functional profiles of gut microbiota from three pig breeds by metagenomic sequencing

**DOI:** 10.3389/fgene.2022.999535

**Published:** 2022-10-14

**Authors:** Taojie Xu, Haichao Sun, Lanlan Yi, Minghua Yang, Junhong Zhu, Ying Huang, Hongbin Pan, Honghui Li, Weizhen Li, Hongye Zhao, Hongjiang Wei, Sumei Zhao

**Affiliations:** ^1^ Yunnan Key Laboratory of Animal Nutrition and Feed Science, Yunnan Agricultural University, Kunming, China; ^2^ Yunnan Province Key Laboratory for Porcine Gene Editing and Xenotransplantation, Kunming, China; ^3^ College of Veterinary Medicine, Yunnan Agricultural University, Kunming, China

**Keywords:** pigs, microbial communities, metagenomic approach, genetic makeup, carbohydrate metabolism, antibiotic resistance genes

## Abstract

To investigate the difference of microbial communities among Diannan small-ear (DNSE), Dahe black (DHB) and Yorkshire (YS) pigs, we compared the microbial taxonomic and functional composition using a metagenomic approach. A total of 1,002,362 non-redundant microbial genes were identified, DHB and YS pigs had more similar genetic makeup compared with DNSE pigs. *Bacteroidetes*, *Firmicutes* and *Spirochetes* were the three most abundant phyla for all pig breeds, and DNSE pigs had a higher abundance of *Prevotella* genus than DHB and YS pigs. The functional profiles varied among the three pig breeds, DNSE pigs had more active carbohydrate metabolism and more abundant antibiotic resistance genes than the other two pig breeds. Moreover, we found that peptide and macrolide resistances genes in DNSE pigs were more abundant than that in DHB pigs (*p* < 0.05). This study will help to provide a theoretical basis for the development of native pig breeds in Yunnan Province, China.

## Introduction

China’s pig production ranks first in the world (Zhang et al., 2020), and it has rich resources of pig breeds, especially the native breeds distributed throughout the country (Wang et al., 2016), which accounted for almost one third of all pig breeds in the world (Yang et al., 2003). Diannan small-ear (DNSE) pigs and Dahe pigs are two typical native breeds that are raised in southern and southwest areas of Yunnan Province, China, respectively (Jiang et al., 2011; Huo et al., 2015). Compared with the foreign breeds such as Yorkshire pigs and Duroc pigs, they have more fat deposition, better meat quality but lower growth rate (Wang et al., 2017; Wu et al., 2020). Dahe black (DHB) pigs are a crossbreed using the Duroc × Dahe breeding scheme through five generations of selection (Jiang et al., 2011), which had high intramuscular fat (IMF) (5.24%) and excellent consumer acceptance (Shi et al., 2019).

The microbial communities in the pig gut perform a variety of beneficial functions and play important roles in maintaining host health (Fouhse et al., 2016; Guevarra et al., 2019). It is comprised of diverse populations of bacteria and other microorganisms, and its components are determined by many factors (Bergamaschi et al., 2020). It is well known that the farm management practices and diets are important aspects of agricultural animal production that could influence gut microbial diversity (Cotillard et al., 2013). The inappropriate use of antibiotics can cause imbalance of gut microbiota, and induce an increased antibiotic resistance in this organisms (Guo et al., 2021). In addition, the host genetics are also a crucial determinant, it was widely reported that the microbial communities have a certain degree of breed specificity (Pajarillo et al., 2015; Sun et al., 2022).

Up to now, there has been no studies comparing the gut microbial diversity among Chinese native pig breeds, foreign pig breeds and hybrids of the two pig breeds. In the present study, we collected the fecal samples from DNSE, DHB, and YS pigs, and established the gut microbial gene catalogue by Illumina-based metagenomic sequencing. Then we compared the taxonomic and functional profiles of the fecal samples, so as to investigate the influence of gut microbiota on hosts and its potential mechanisms.

## Materials and methods

### Animal feeding and sample collection

In this study, three DNSE, DHB and YS pigs in the experimental pig farm of Yunnan Agricultural University were used. Each pig was raised in a single pen and all pigs were fed the same basic diet without any antibiotics. When pigs reached 160 days of age, fresh fecal samples were collected from every animal and put into liquid nitrogen for storage. The animal study was approved by the Animal Care and Use Committee of Yunnan Agricultural University.

### Library construction and sequencing

Total DNA was extracted from each fecal sample, and a total amount of 1 μg DNA per sample was used as input material for the library construction. Sequencing libraries were generated using NEBNext^®^ Ultra™ DNA Library Prep Kit for Illumina (NEB, United States) following manufacture’s recommendations and index codes were added to attribute sequences to each sample. Briefly, the DNA sample was fragmented by sonication to a size of 350 bp, then DNA fragments were end-polished, A-tailed, ligated with the full-length adaptor and amplified by PCR. At last, PCR products were purified using AMPure XP system, analyzed for size distribution by Aglient 2100 Bioanalyzer and quantified using real-time PCR. The clustering of the index-coded samples was performed on a cBot Cluster Generation System according to the manufacture’s instructions. After cluster generation, the library preparations were sequenced on an Illumina HiSeq platform to generate the paired-end reads.

### Construction of gene catalogue

The raw sequencing data was processed using Readfq (v8) to acquire the clean data, and clean reads were align to pig genome with SOAPaligner (v2.21) to discard host sequences. Then, all reads were assembled using SOAP *denovo* (v2.04) (Luo et al., 2012), the assembled Scaftigs (≥500 bp) were predicted open reading frames (ORFs) by MetaGeneMark (v2.10) (Nielsen et al., 2014). Subsequently, a non-redundant gene set was constructed by pair-wise comparison of all genes using CD-HIT (v4.6.6) (Fu et al., 2012), genes with reads less than 2 were selected for subsequent analysis (Gao et al., 2021). All data generated for this study were deposited in the National Center for Biotechnology Information (NCBI) Sequence Read Archive (SRA) under BioProject ID PRJNA872826. The abundance of each gene was calculated by the following equation:

$$G_{k}=\frac{r_{k}}{L_{k}}∙\frac{1}{\overset{n}{\underset{i=1}{\sum }}\frac{r_{i}}{L_{i}}}$$

where *r* was the read number of gene, *L* was the length of gene.

### Taxonomic annotation

All genes in our catalogue were translated to amino acid sequences and aligned to the NCBI-NR database (v20200604) using DIAMOND (v0.9.9) (e-value ≤ 1e-5) (Buchfink et al., 2015). For the alignment results of each gene, results with e-value ≤ 10*minimum e-value were selected for further analysis. Then, the lowest common ancestor (LCA) algorithm of MEGAN4 was used to sort genes into taxonomic groups with the default parameters (Joynson et al., 2017). The abundance of a taxon was calculated as the sum of the abundances of matched genes.

### Functional annotation

To investigate the functional composition, putative amino acid sequences were aligned against protein sequences from Kyoto Encyclopedia of Genes and Genomes (KEGG) database (v82) and Carbohydrate-Active Enzymes (CAZy) database (v6.0) using DIAMOND (e-value ≤ 1e-5). Each protein was assigned to each database by the highest scoring annotated hit(s) containing at least one HSP scoring >60 bits (Bäckhed et al., 2015). Finally, we aligned the protein sequences against reference sequences from Comprehensive Antibiotic Resistance Database (CARD, v2.0.1) by using Resistance Gene Identifier (RGI) (v5.1.0). The functional abundances were calculated as the sum of the abundances of genes annotated to different functional groups.

### Statistical analysis

Analysis of variance (ANOVA, Duncan’s multiple range test) was performed using agricolae package in R (v4.1.2), significant differences between each two groups were indicated by *p < 0.05 and **p < 0.01. The Venn diagram heat map and chord diagram were generated using the VennDiagram, pheatmap and circlize packages in R, respectively.

## Results

### Construction of gene catalogue

We conducted a sequence analysis after Illumina sequencing, the specific information was shown in Supplementary Table S1. In brief, an average of 6.4 Gb (ranging between 6.09 and 6.77 Gb) of sequence was generated for each sample (Supplementary Table S1). In total, we obtained 57.49 Gb of clean reads after removing the host sequences. Then, we obtained 1.23 million Scaftigs with an average length of 1091.34 bp (ranging between 682.26 and 1197.39 bp) by metagenomic assembly (Supplementary Table S2). At last, a gene catalogue contained 1,002,362 non-redundant genes were produced for subsequent analysis.

### Genetic makeup of gut microbiota

**FIGURE 1:**
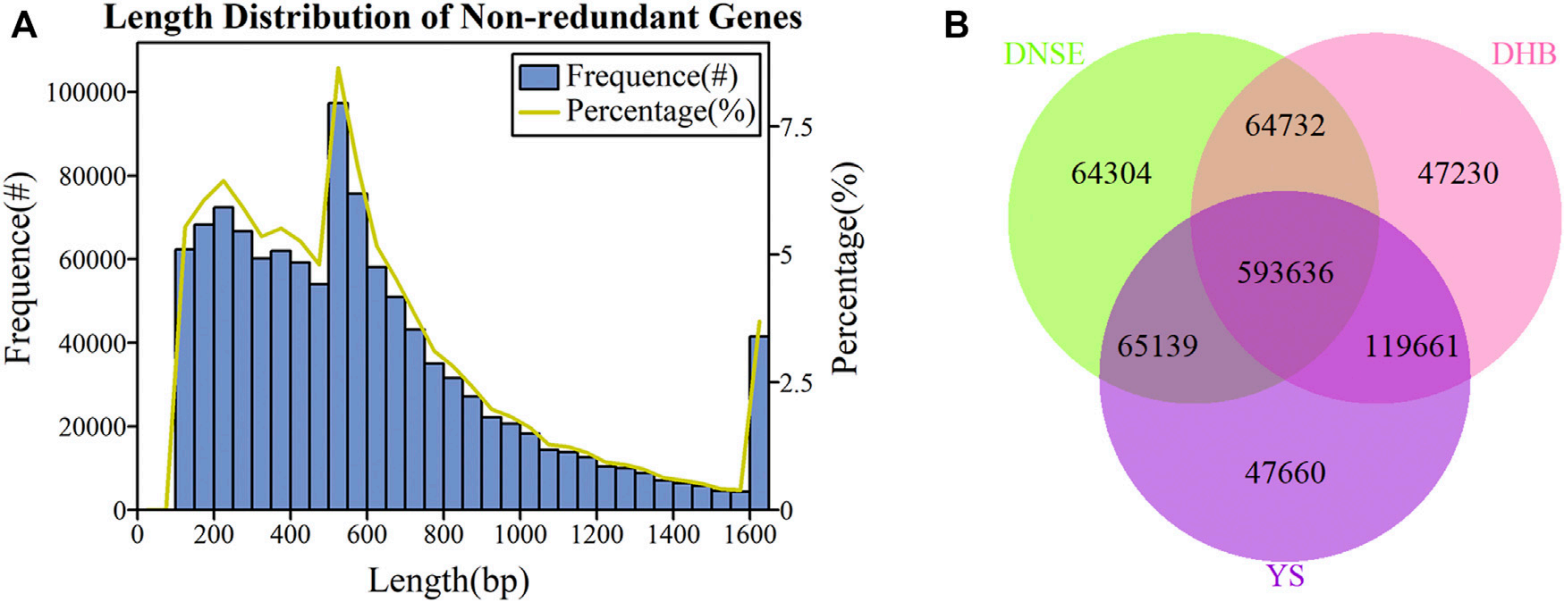
**(A)** Length distribution of non-redundant genes. **(B)** Venn diagram showing the number of shared and unique microbial non-redundant genes among DNSE, DHB and YS pigs.

As exhibited in Figure 1A, the majority of non-redundant genes had lengths less than 600 bp, of which the most genes were between 500 and 550 bp. Moreover, when the gene exceeded 600 bp, the gene number gradually decreased as its length became longer. To investigate the effect of sample size on gene diversity, we constructed the rarefaction curves of core and pan genes. As indicated in Supplementary Figure S1, the core gene gradually decreased and the pan gene gradually increased with the increase of sample size, and they tend to be stable when all nine samples were used.

We identified 787,811, 825,259 and 826,096 microbial genes from fecal samples of DNSE, DHB and YS pigs, respectively, the gene number of each sample was shown in Supplementary Figure S2. More specifically, there were 593,636 common genes present in all pig breeds and 64,304, 47,230, 47,660 unique genes present in DNSE, DHB and YS pigs, respectively (Figure 1B). Besides, DHB and DNSE pigs shared 658,368 common genes, DNSE and YS pigs shared 658,775 common genes, DHB and YS pigs shared 713,297 common genes (Supplementary Figure S3). Hence, it could be demonstrated that the genetic makeup of DHB and YS pigs was more similar compared with DNSE pigs.

### Taxonomic composition of gut microbiota

Among the 1,002,362 non-redundant genes in the gene catalogue, 778,380 genes were assigned to the NCBI-NR database, the matching rate was 77.65% (Supplementary Table S3). Among all matched genes, 665,515 (85.50%), 630,332 (80.98%), 587,054 (75.42%), 583,941 (75.02%), 508,049 (65.27%), 485,631 (62.39%) and 357,432 (45.92%) genes were assigned to kingdom, phylum, class, order, family, genus and species levels, respectively. Finally, we identified a rich taxonomic structure, including four kingdoms, 62 phyla, 85 classes, 188 orders, 408 families, 1509 genera and 6008 species.

**FIGURE 2:**
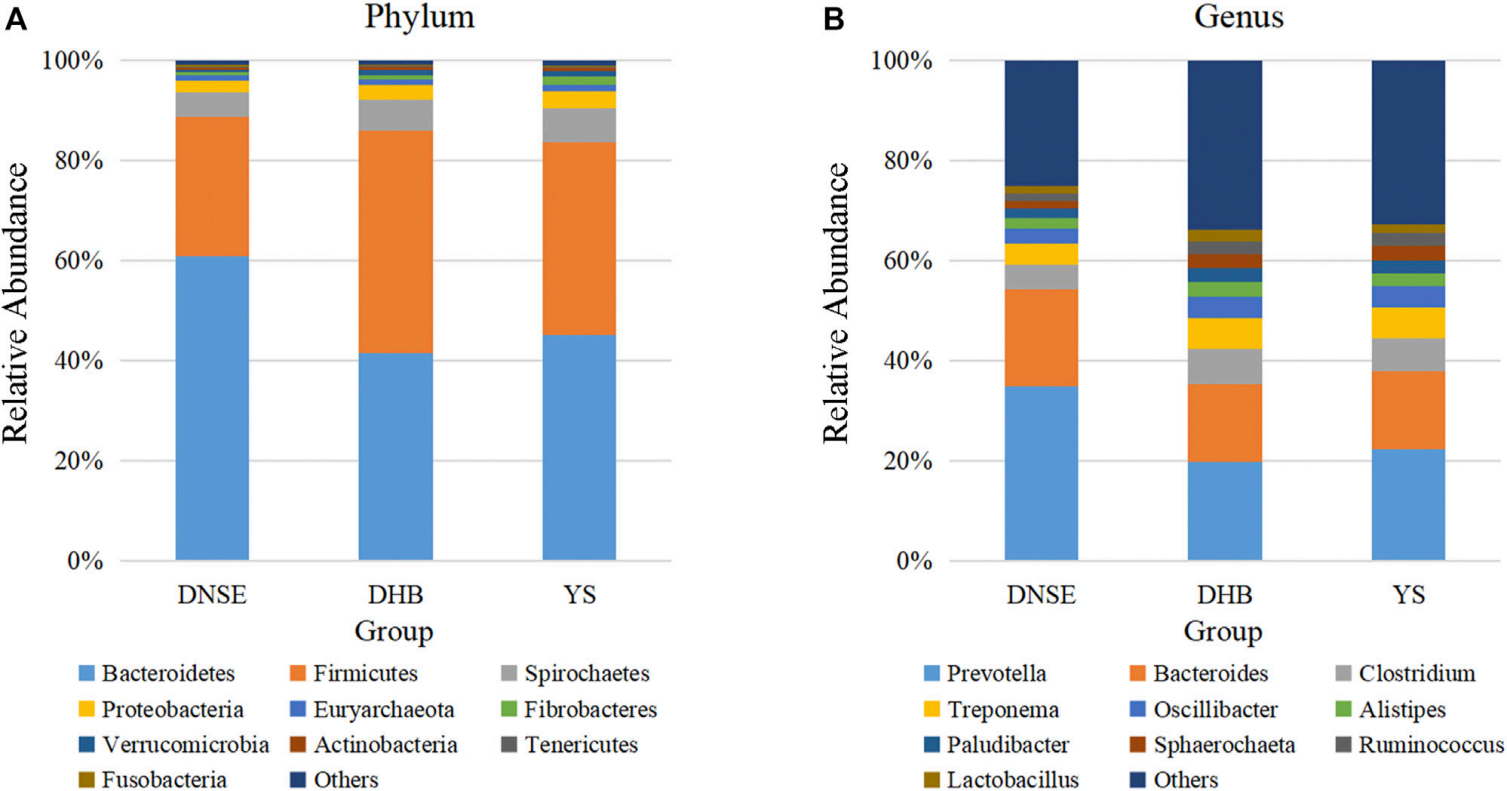
Gut microbial taxonomic composition at the phylum **(A)** and genus **(B)** levels.

At the phylum level, *Bacteroidetes and Firmicutes* were the two dominant phyla for all pig breeds, their total relative abundance were more than 80% (Figure 2A). For DNSE pigs, the most abundant phylum was *Bacteroidetes*; For DHB and YS pigs, the most abundant phylum was *Bacteroidetes.* Moreover, *Spirochaetes* and *Proteobacteria* were the third and fourth most abundant phyla for all pig breeds, respectively. The relative abundance of *Bacteroidetes* in DNSE pigs was higher than that in DHB and YS pigs, while the relative abundances of *Firmicutes*, *Spirochaetes* and *Proteobacteria* in DNSE pigs were lower than that in DHB pigs.

At the genus level, *Prevotella* and *Bacteroides* were found to be the two main genera for all pig breeds, followed by *Clostridium* and *Treponema* (Figure 2B). The relative abundances of *Prevotella* and *Bacteroides*s in DNSE pigs were higher than that in DHB and YS pigs, while the relative abundances of *Clostridium* and *Treponema* in DNSE pigs were lower than that in DHB and YS pigs.

### Kyoto encyclopedia of genes and genomes annotation of gene catalogue

**FIGURE 3:**
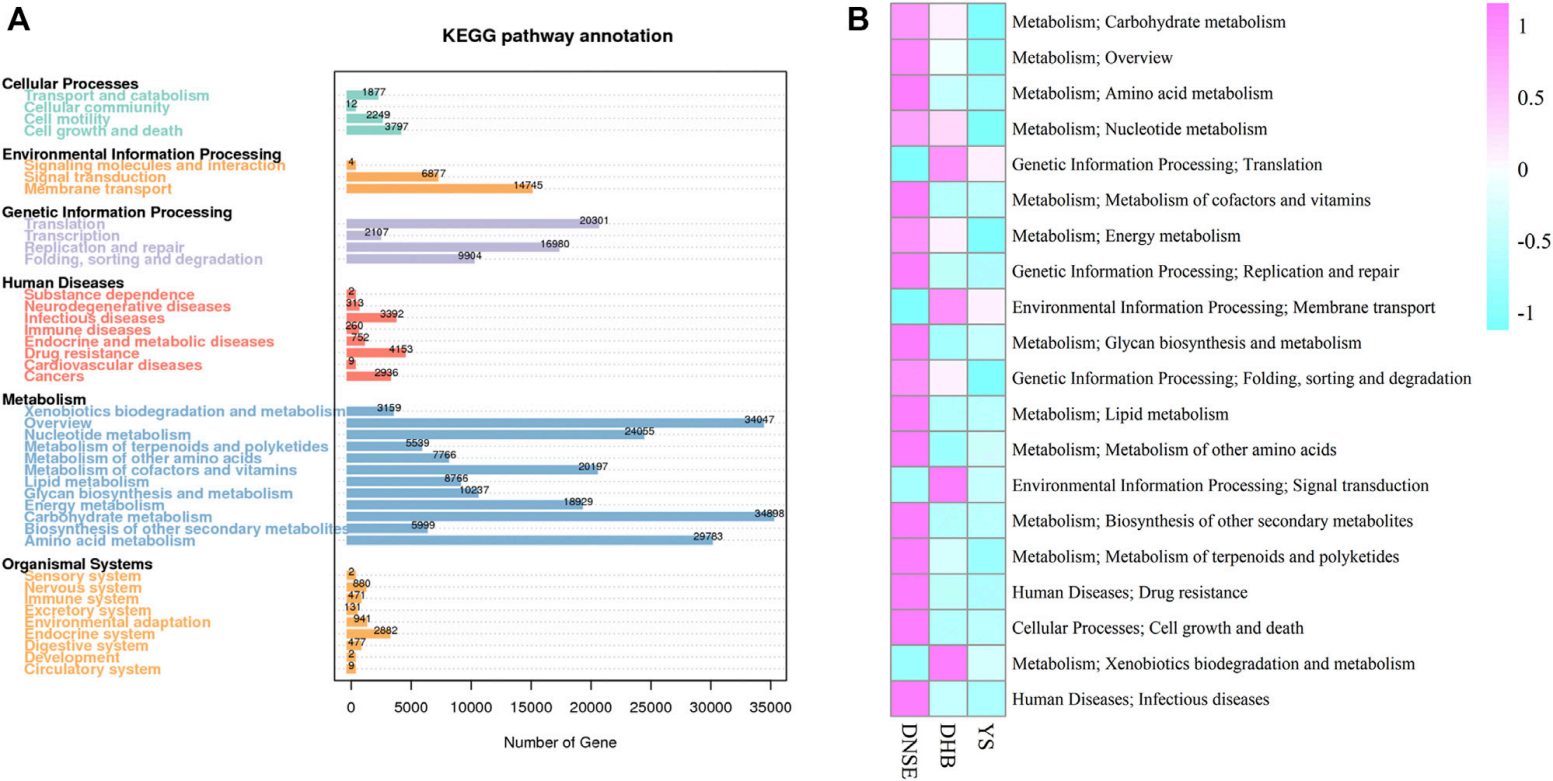
**(A)** Number of non-redundant genes assigned to KEGG pathways. **(B)** The 20 most abundant KEGG pathways.

Among the 1,002,362 non-redundant genes in the gene catalogue, 537,626 genes were assigned to KEGG database, the matching rate was 53.64% (Table S4). In total, we obtained 3800 KEGG orthologys (KOs) and 580 KEGG pathways. As shown in Figure 3A, most genes were assigned to carbohydrate metabolism (34,898) and overview (34,047), and 4,153 genes were assigned to drug resistance. Among the top 20 most abundant pathways, 16 pathways were more abundant in DNSE pigs than in DHB and YS pigs (Figure 3B). In line with the matched genes, carbohydrate metabolism and overview were the two most abundant pathways. Furthermore, we found that the abundances of carbohydrate metabolism and drug resistance in DNSE pigs were the highest, followed by DHB pigs, and the lowest in YS pigs.

### Carbohydrate-active enzymes profiles in the pig gut

**FIGURE 4:**
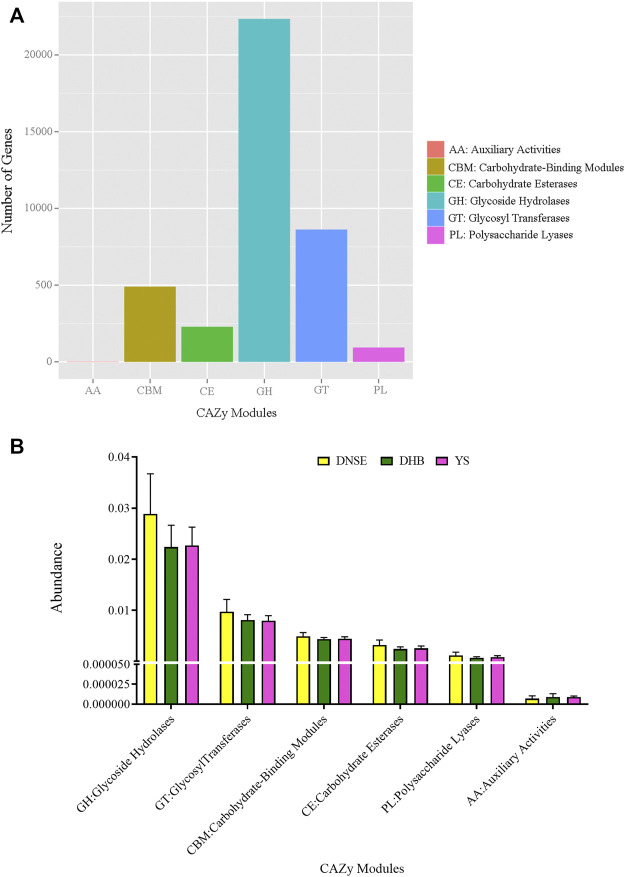
**(A)** Number of non-redundant genes assigned to CAZy modules. **(B)** Abundances of CAZy modules. Error bars represents SD of three replicates.

Among the 1,002,362 non-redundant genes in the gene catalogue, 36,429 genes were assigned to CAZy database, the matching rate was 3.63% (Supplementary Table S4). In total, we obtained 224 CAZy families covering six modules. The genes assigned to glycoside hydrolases (GH) were the most, followed by glycosyl transferases (GT) and carbohydrate-binding modules (CBM), while the genes assigned to auxiliary activities (AA) were few (Figure 4A). As shown in Figure 4B, the abundances of GH, GT, CBM, carbohydrate esterases (CE) and polysaccharide lyases (PL) in DNSE pigs were higher than that in DHB and YS pigs, while the abundance of AA in DNSE pigs was lower than that in DHB and YS pigs.

**FIGURE 5:**
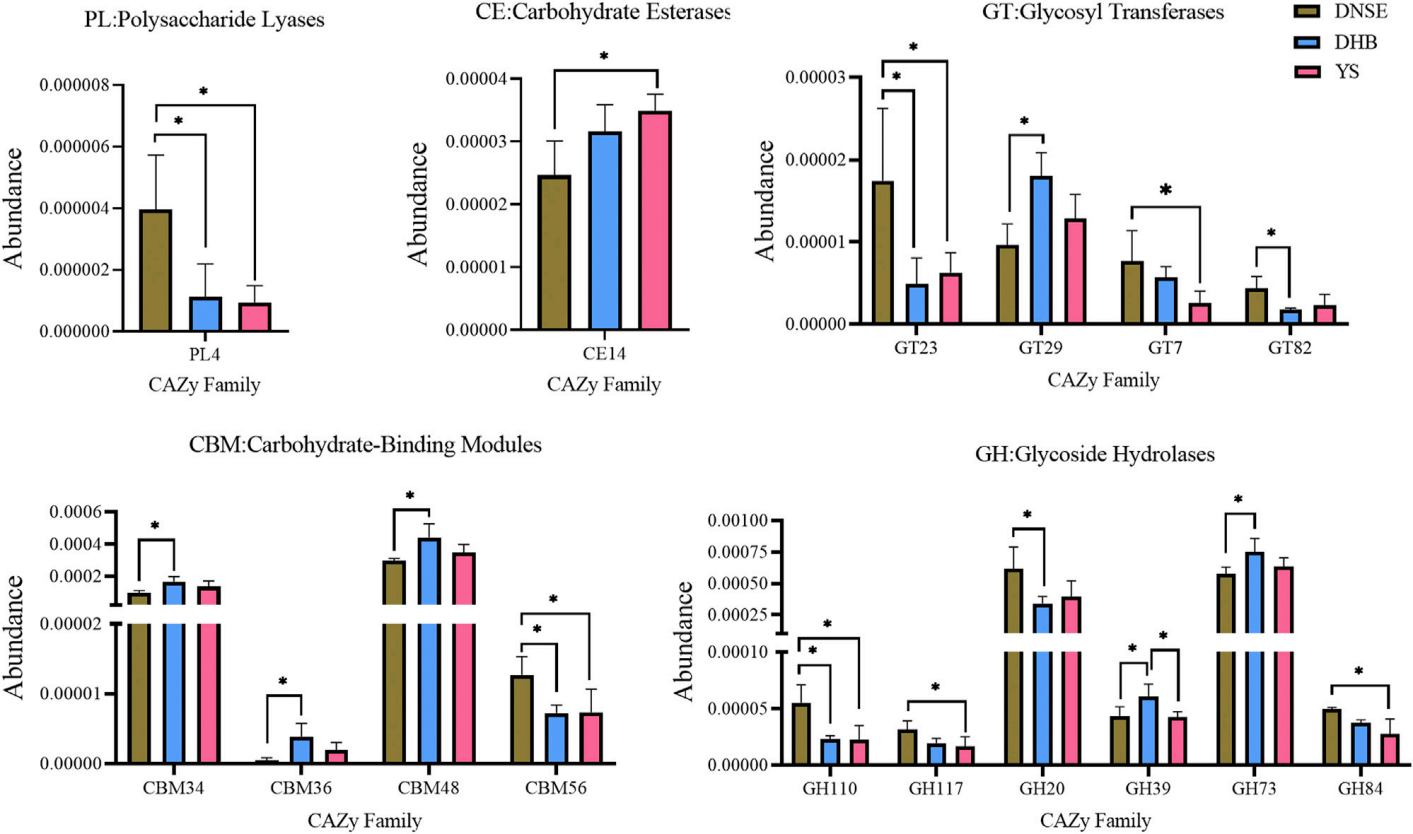
CAZy families with significant differences among three pig breeds. Error bars represents SD of three replicates, *p < 0.05.

The analysis of variance showed that the abundances of 16 CAZy families covering five modules varied significantly among the three pig breeds (Figure 5). Compared with DHB pigs, DNSE pigs had a higher abundance of PL4, GT23, GT82, CBM56, GH110, GH20, and a lower abundance of GT29, CBM34, CBM36, CBM48, GH39 and GH73 (*p* < 0.05). Compared with YS pigs, DNSE pigs had a higher abundance of PL4, GT23, GT7, CBM56, GH110, GH117, GH84, and a lower abundance of CE14 (*p* < 0.05). Furthermore, we found that GH39 was the single family with significant difference between DHB and YS pigs, and its abundance in DHB pigs was higher (*p* < 0.05).

### Antibiotic resistance genes in the pig gut

**FIGURE 6:**
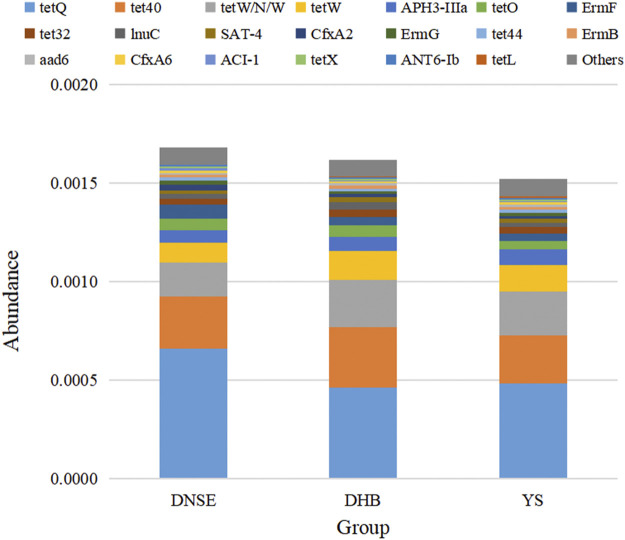
The 20 most abundant ARGs.

Among the 1,002,362 non-redundant genes in the gene catalogue, 126 genes were assigned to CARD database, the matching rate was only 0.01% (Supplementary Table S4). In total, we obtained 87 ARGs which confer resistance to 28 antibiotics. As shown in Figure 6, the total ARG abundance in DNSE pigs was the highest, followed by DHB pigs, and the lowest in YS pigs. Moreover, the four tetracycline resistance genes *tetQ, tet40*, *tetW/N/W* and *tetW* were the most abundant ARGs for all pig breeds, their abundances accounted for more than 50% of the total ARG abundance.

**FIGURE 7:**
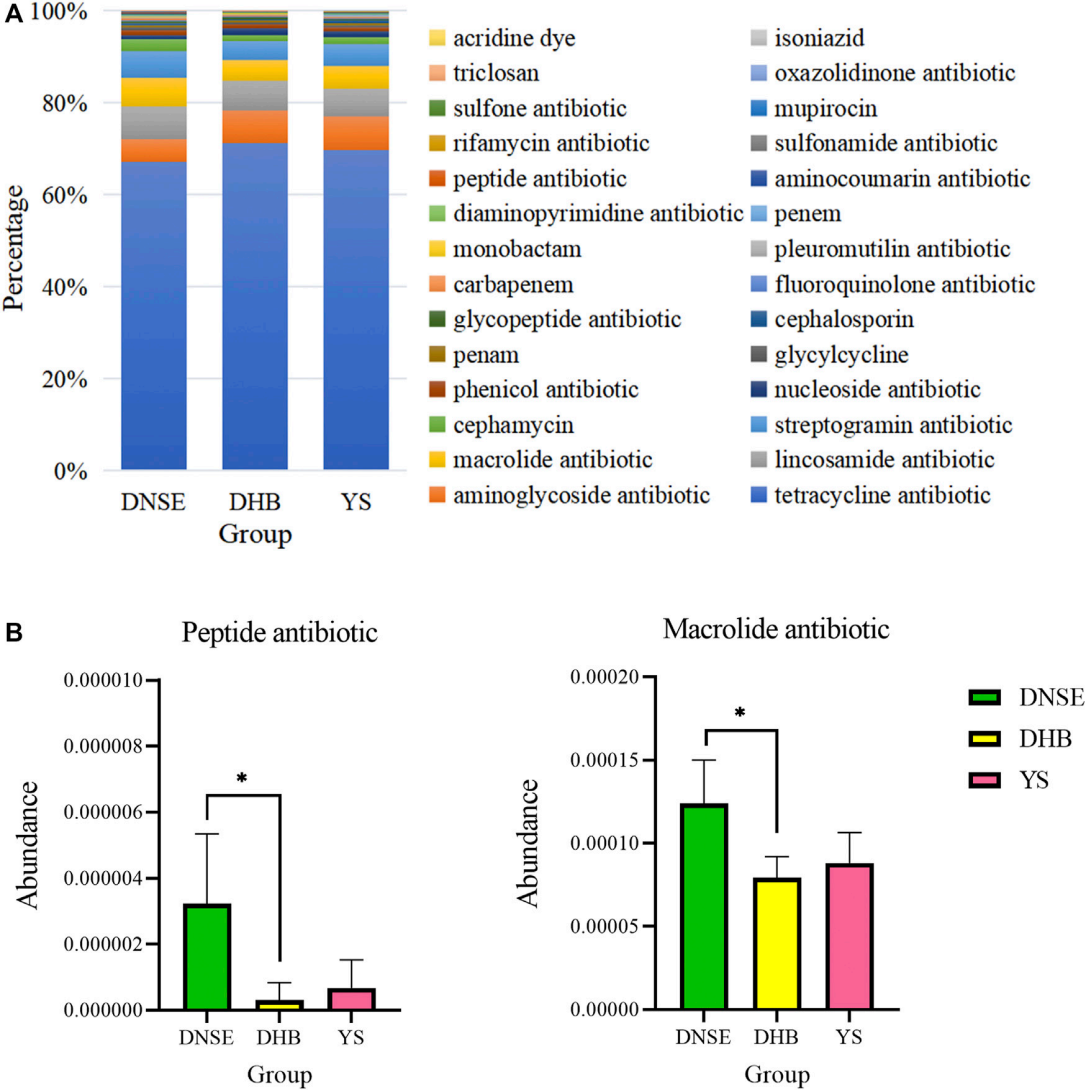
**(A)** Proportions of ARG types. **(B)** ARG types with significant differences among three pig breeds. Error bars represents the SD of three replicates, *p < 0.05.

Among the 28 ARG types, tetracycline resistance genes were the dominant type for all pig breeds, accounting for more than 60% of all ARGs (Figure 7A). In addition to tetracycline resistance genes, the aminoglycoside, lincosamide, macrolide and streptogramin resistance genes were the other main types. The analysis of variance indicated that the abundances of two ARG types varied significantly between DNSE and DHB pigs (Figure 7B). The abundances of peptide and macrolide resistances genes in DNSE pigs were higher than that in DHB pigs (*p* < 0.05).

**FIGURE 8:**
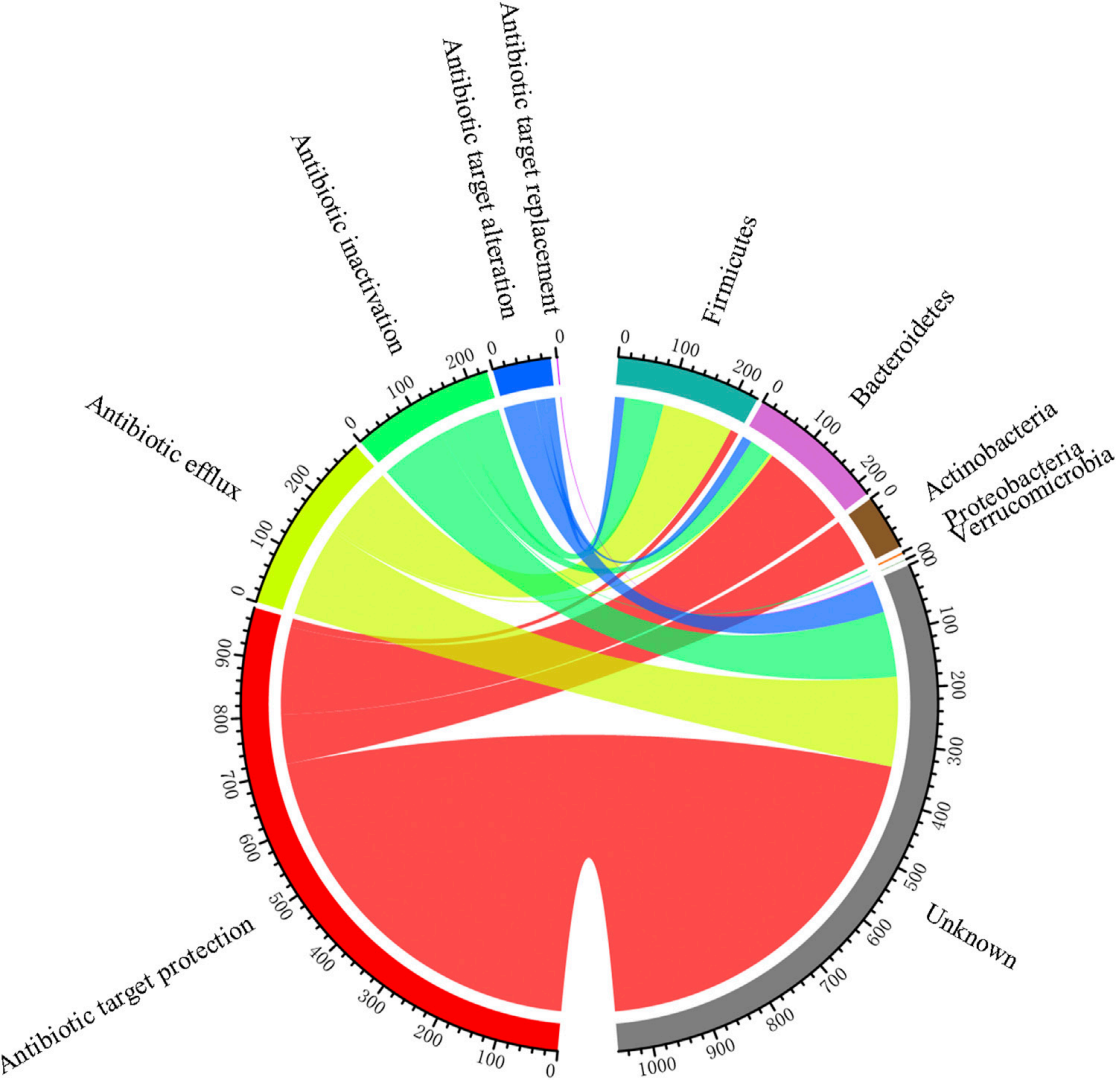
ARG profiles based on their hosts and resistance mechanisms.

The 87 ARGs found in the pig gut had five resistance mechanisms, of which antibiotic target protection was the dominant mechanism, followed by antibiotic efflux (Figure 8). According to the taxonomic information of the non-redundant genes that assigned to CARD database, we investigated the hosts of ARGs. As shown in Supplementary Tables S5, S6, a total of 14 genera were identified to be the hosts of ARGs. At the phylum level, *Firmicutes* and *Bacteroidetes* were the dominant hosts, followed by *Actinobacteria.* At the genus level, *Bacteroides* were the main hosts, followed by *Lachnoclostridium* and *Clostridium.* Besides, as exhibited in Supplementary Table S6, the most abundant genus *Prevotella* had a high abundance of ARGs, antibiotic inactivation and antibiotic target alteration were found to be the resistance mechanisms of this genus.

## Discussion

The host genetics are consider as one important factor that could influence gut microbial diversity (Pajarillo et al., 2015; Sun et al., 2022), genetically related individuals tend to have more similar gut microbial composition than unrelated individuals (Campbell et al., 2012; Hildebrand et al., 2013). In order to explore the influence of host genetics on gut microbial community, we selected three pig breeds raised in the same environment as the subjects. Our results exhibited that DHB and YS pigs had a more similar genetic makeup compared with DNSE pigs, indicating that the Duroc × Dahe breeding scheme resulted in the gut microbiota of native pigs being close to the foreign pigs.

In agreement with many previous studies (Ramayo-Caldas et al., 2016; Holman et al., 2017; Yang et al., 2018), our study showed that the overwhelming majority of phyla in pig feces were *Bacteroidetes* and *Firmicutes*. *Prevotella* are usually found to be the most abundant genus in pig feces (Ramayo-Caldas et al., 2016; Holman et al., 2017), which is consistent with the result in this study. The second abundant genus varies in different studies, we found that *Bacteroides* were the second abundant genus in pig feces, but other studies reported that *Treponema* (Holman et al., 2017) or *Roseburia* (Ramayo-Caldas et al., 2016) were the second abundant genus in pig feces. The differences in microbial composition may be due to the different growth environment and dietary composition (Cotillard et al., 2013).

*Prevotella* is essential for a healthy gut microbial community, which can improve digestion of dietary protein and carbohydrates (Dao et al., 2021). However, the increased abundance of *Prevotella* at mucosal sites will induce localized and systemic disease such as rheumatoid arthritis, metabolic disorders and low-grade systemic inflammation (Larsen, 2017). The higher abundance of *Prevotella* in DNSE pigs than DHB and YS pigs will improve the host’s digestive capacity, but also increase the risk of host infection. Its important role in the host’s digestive capacity may be a underlying factor contributing to the higher IMF in DNSE pigs.

Bacteria are the main hosts of ARGs, the bacterial community structure can determine the ARG profiles in the environment (Sun et al., 2016). The results of KEGG and CARD database annotations demonstrated that DNSE pigs had higher ARG abundance than DHB and YS pigs, which may be due to differences in gut microbial composition. *Prevotella* is a common host for ARGs (Sherrard et al., 2014), its strong antibiotic resistance was well confirmed in our results. Therefore, the higher ARG abundance of DNSE pigs may be partly due to the higher abundance of *Prevotella* than the other two pig breeds.

Carbohydrates are high in diets and are considered as primary energy source for maintenance, growth, and production in animals (Nafikov and Beitz, 2007). The intake of diet carbohydrate was inversely associated with risk of overweight or obesity (Merchant et al., 2009). Carbohydrate-active enzymes are a large class of enzymes that catalyze the breakdown, biosynthesis or modification of carbohydrates and glycoconjugates. These enzymes in gut microbiota could enhance the digestion and absorption efficiency of dietary carbohydrates for the host. The results of the KEGG and CAZy database annotations indicated that the gut microbiota of DNSE pigs was more active in carbohydrate metabolism than DHB and YS pigs, which may be one of the reasons for more fat deposition and higher IMF in DNSE pigs.

The tetracycline resistance genes can be divided into ribosome protection protein, efflux protein and inactivating enzyme according to the resistance mechanism. Ribosome protection proteins represent an important class that promote tetracycline resistance in both Gram-positive and -negative species by binding to the ribosome and chasing the drug from its binding site (Arenz et al., 2015). Our results showed that three ribosome protection proteins (*tetQ*, *tetW/N/W* and *tetW*) and one efflux protein (*tet40*) were the most abundant ARGs, suggesting that ribosome protections protein were the major class of tetracycline resistance genes, followed by efflux proteins.

## Conclusion

In conclusion, the taxonomic and functional profiles of gut microbiota were associated with pig breeds. DNSE pigs had more active carbohydrate metabolism and more abundant ARGs than the other two pig breeds. DNSE pigs had significantly stronger resistance to peptide and macrolide antibiotics than DHB pigs. The higher IMF and stronger antibiotic resistance of DNSE pigs may be related to the higher abundance of *Prevotella* than DHB and YS pigs.

## Data Availability

The data presented in the study are deposited in the NCBI SRA, accession number PRJNA872826.
