# Supplementary material for: Comparing the taxonomic and functional profiles of gut microbiota from three pig breeds by metagenomic sequencing
Source: Front Genet. 2022 Oct 14;13:999535. doi: 10.3389/fgene.2022.999535 (PMC9614230; doi:10.3389/fgene.2022.999535)
Supplement: Supplementary file 1 [file DataSheet1.DOCX]

**Supplementary Material**

**
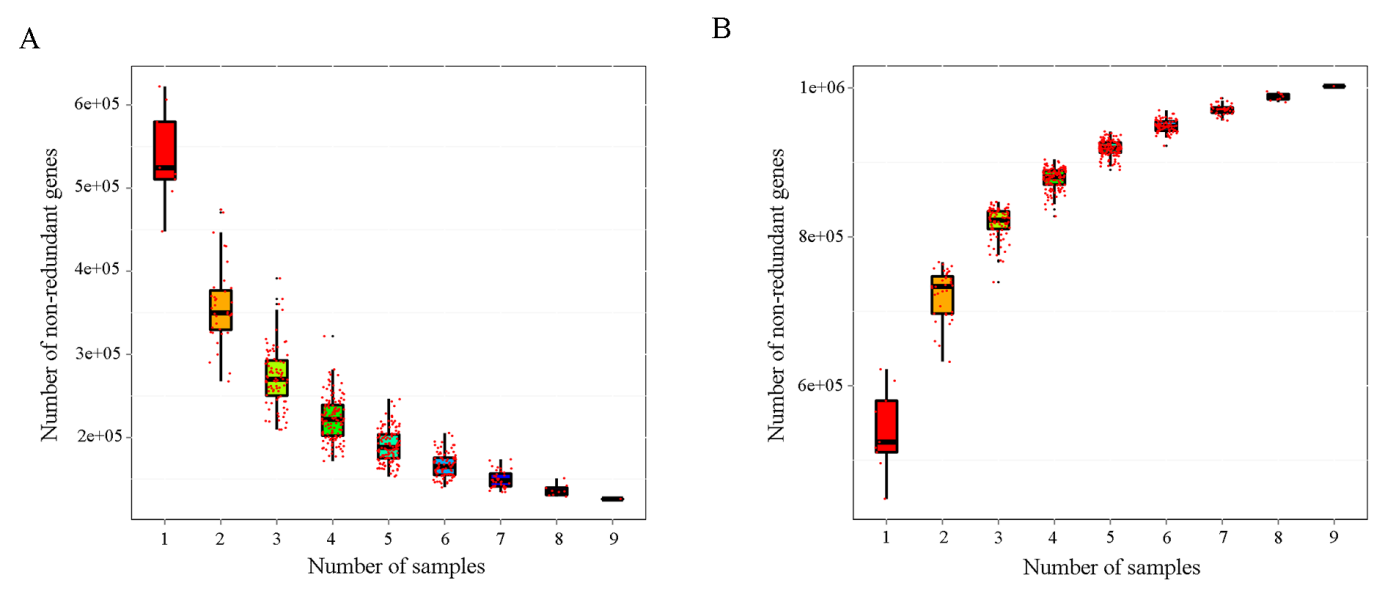
**

**Figure S1|** The rarefaction curves of core **(A)** and pan **(B)** genes.

**
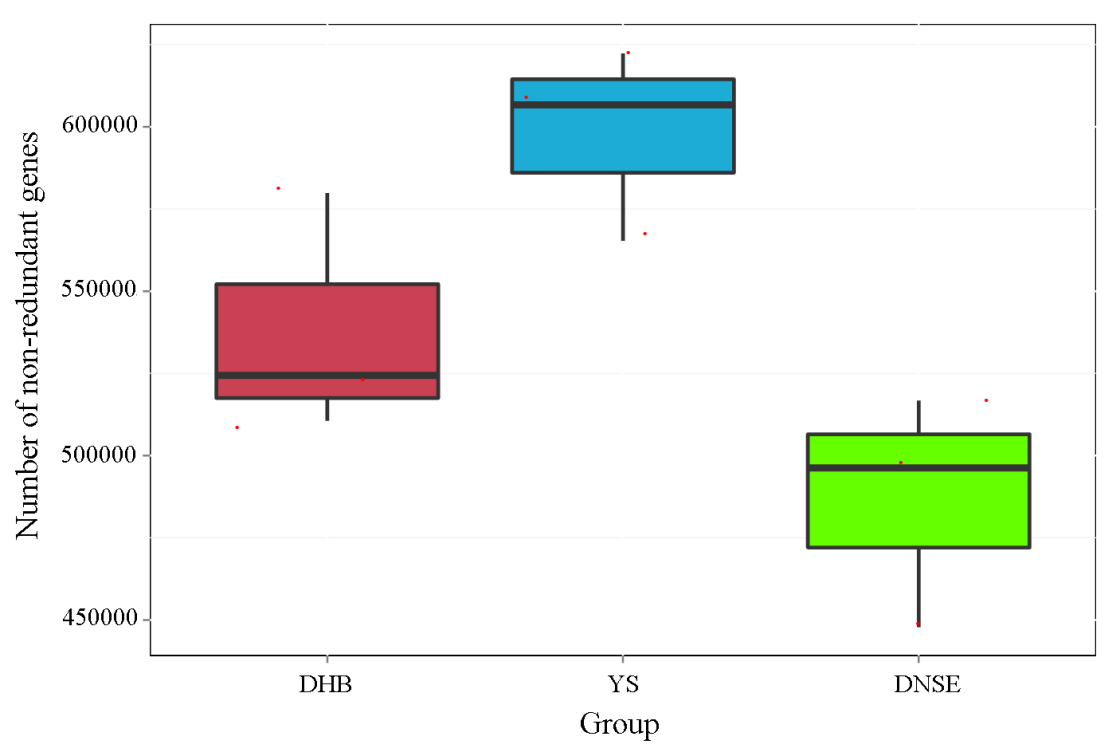
**

**Figure S2|** The number of non-redundant genes in fecal samples of the three pig breeds.

**
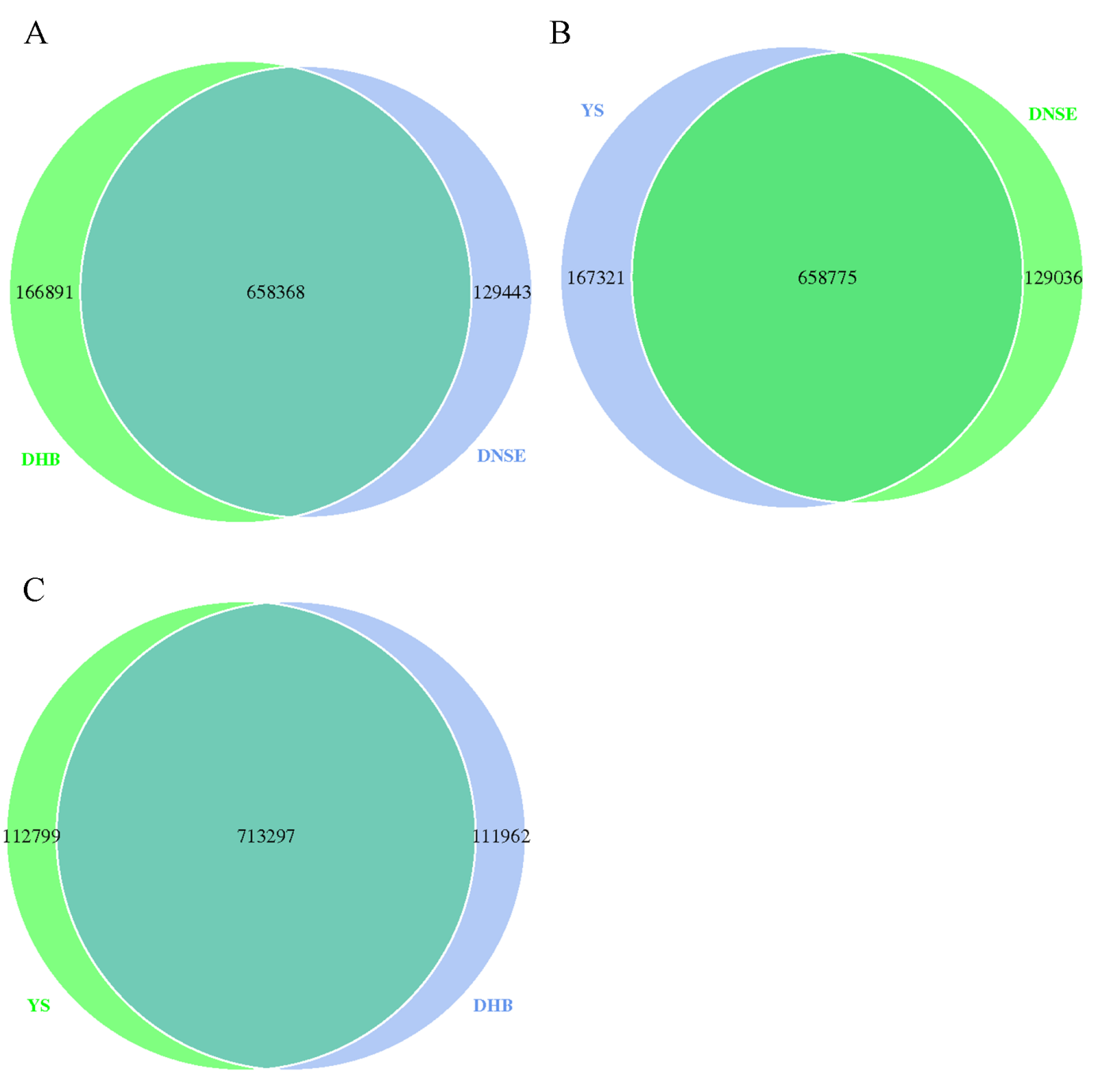
**

**Figure S3|** Venn diagram showing the number of shared and unique microbial non-redundant genes among each two pig breeds.

**Table S1|** Summary of sequence analysis after Illumina sequencing.

| Sample | Raw reads | Raw bases (Mbp) | Clean bases (Mbp) | Clean GC (%) | Effective (%) | Nonhost bases (Mbp) |
| --- | --- | --- | --- | --- | --- | --- |
| DNSE.1 | 42,479,152 | 6,371.87 | 6,363.75 | 46.95 | 99.87 | 6,361.70 |
| DNSE.2 | 40,582,880 | 6,087.43 | 6,080.95 | 47.14 | 99.89 | 6,079.42 |
| DNSE.3 | 43,360,400 | 6,504.06 | 6,488.49 | 46.97 | 99.76 | 6,485.88 |
| DHB.1 | 42,931,874 | 6,439.78 | 6,432.92 | 47.06 | 99.89 | 6,432.74 |
| DHB.2 | 42,630,530 | 6,394.58 | 6,385.84 | 48.47 | 99.86 | 6,385.26 |
| DHB.3 | 40,887,908 | 6,133.19 | 6,123.36 | 45.9 | 99.84 | 6,122.14 |
| YS.1 | 44,465,584 | 6,669.84 | 6,658.07 | 46.82 | 99.82 | 6,657.12 |
| YS.2 | 41,627,802 | 6,244.17 | 6,233.68 | 48.39 | 99.83 | 6,225.24 |
| YS.3 | 45,103,514 | 6,765.53 | 6,750.27 | 47.33 | 99.78 | 6,743.70 |

Nonhost bases: The clean bases after removing the host sequences; Clean GC : The content of G and C in clean bases. Effective: The ratio of clean bases to raw bases.

| Sample | Scaftig number | Total length (Mbp) | Average length (bp) | Max length (bp) | N50 (bp) | N90 (bp) |
| --- | --- | --- | --- | --- | --- | --- |
| DNSE.1 | 128,013 | 147.13 | 1,149.30 | 216,365 | 1,248 | 579 |
| DNSE.2 | 118,147 | 141.47 | 1,197.39 | 229,583 | 1,326 | 585 |
| DNSE.3 | 137,365 | 158.43 | 1,153.34 | 151,314 | 1,261 | 584 |
| DHB.1 | 136,934 | 157.94 | 1,153.41 | 109,967 | 1,226 | 578 |
| DHB.2 | 120,594 | 135.01 | 1,119.58 | 160,501 | 1,195 | 577 |
| DHB.3 | 117,782 | 133.74 | 1,135.47 | 136,072 | 1,221 | 578 |
| YS.1 | 135,087 | 149.37 | 1,105.75 | 112,741 | 1,174 | 578 |
| YS.2 | 122,621 | 135.82 | 1,107.64 | 214,020 | 1,175 | 572 |
| YS.3 | 129,877 | 144.06 | 1,109.22 | 126,670 | 1,159 | 574 |
| NOVO MIX | 82,564 | 56.33 | 682.26 | 5,528 | 658 | 523 |

**Table S2|** Summary of metagenomic assembly.

NOVO MIX: The mixed assembly of unused reads in all samples during assembly; N50, N90: Sort all Scaftigs by length, and then add them from long to short. When the sum reaches 50% or 90% of the total length, the length represents N50 or N90.

**Table S3|** Summary of taxonomic annotation.

| Taxonomic level | Gene number | Matching rate (%) | Proportion (%) |
| --- | --- | --- | --- |
| Kingdom | 665,515 | 66.39 | 85.50 |
| Phylum | 630,332 | 62.88 | 80.98 |
| Class | 587,054 | 58.57 | 75.42 |
| Order | 583,941 | 58.26 | 75.02 |
| Family | 508,049 | 50.69 | 65.27 |
| Genus | 485,631 | 48.45 | 62.39 |
| Species | 357,432 | 35.66 | 45.92 |
| NCBI-NR database | 778,380 | 77.65 | 100 |

Gene number: The number of genes that assigned to each taxonomic level; Matching rate: The ratio of matched genes at each taxonomic level to all genes in the gene catalogue; Proportion: The proportion of matched genes at each taxonomic level among all genes that assigned to NCBI-NR database.

**Table S4|** Summary of functional annotation.

| Database name | Gene number | Matching rate (%) |
| --- | --- | --- |
| KEGG | 537,626 | 53.64 |
| CAZy | 36,429 | 3.63 |
| CARD | 126 | 0.01 |

Gene number: The number of genes that assigned to each database. Matching rate: The ratio of matched genes to all genes in the gene catalogue.

**Table S5|** ARG abundances based on microbial phylum and resistance mechanisms in all fecal samples.

| Phylum | ATP | AE | AI | ATA | ATR | Total |
| --- | --- | --- | --- | --- | --- | --- |
| *Firmicutes* | 1.4e-04 | 1.2e-03 | 6.4e-04 | 1.6e-04 | 0 | 2.1e-03 |
| *Bacteroidetes* | 1.4e-03 | 5.1e-05 | 3.6e-04 | 1.6e-04 | 0 | 2.0e-03 |
| *Actinobacteria* | 8.1e-04 | 0 | 0 | 0 | 0 | 8.1e-04 |
| *Proteobacteria* | 0 | 0 | 2.5e-05 | 2.8e-06 | 0 | 2.8e-05 |
| *Verrucomicrobia* | 0 | 0 | 0 | 4.4e-06 | 0 | 4.4e-06 |
| Unknown | 6.4e-03 | 1.5e-03 | 1.1e-03 | 5.2e-04 | 1.4e-05 | 9.5e-03 |

ATP: antibiotic target protection; AE: antibiotic efflux; AI: antibiotic inactivation; ATA: antibiotic target alteration; ATR: antibiotic target replacement.

| Genus | ATP | AE | AI | ATA | ATR | Total |
| --- | --- | --- | --- | --- | --- | --- |
| *Bacteroides* | 4.2e-04 | 2.8e-05 | 1.0e-04 | 1.5e-04 | 0 | 7.0e-04 |
| *Lachnoclostridium* | 0 | 0 | 7.2e-05 | 0 | 0 | 7.2e-05 |
| *Clostridium* | 5.2e-05 | 9.1e-06 | 2.0e-06 | 0 | 0 | 6.3e-05 |
| *Exiguobacterium* | 0 | 0 | 2.8e-05 | 0 | 0 | 2.8e-05 |
| *Campylobacter* | 0 | 0 | 2.2e-05 | 0 | 0 | 2.2e-05 |
| *Prevotella* | 0 | 0 | 4.7e-06 | 9.0e-06 | 0 | 1.4e-05 |
| *Staphylococcus* | 0 | 0 | 9.0e-06 | 0 | 0 | 9.0e-06 |
| *Akkermansia* | 0 | 0 | 0 | 4.4e-06 | 0 | 4.4e-06 |
| *Oscillibacter* | 0 | 0 | 3.6e-06 | 0 | 0 | 3.6e-06 |
| *Ruminococcus* | 0 | 3.4e-06 | 0 | 0 | 0 | 3.4e-06 |
| *Desulfovibrio* | 0 | 0 | 0 | 2.8e-06 | 0 | 2.8e-06 |
| *Bacillus* | 0 | 0 | 1.7e-06 | 0 | 0 | 1.7e-06 |
| *Caldicellulosiruptor* | 0 | 0 | 1.6e-06 | 0 | 0 | 1.6e-06 |
| *Blautia* | 0 | 0 | 1.5e-06 | 0 | 0 | 1.5e-06 |
| Unknown | 8.3e-03 | 2.7e-03 | 1.8e-03 | 6.8e-04 | 1.4e-05 | 1.4e-02 |

**Table S6|** ARG abundances based on microbial genus and resistance mechanisms in all fecal samples.
